# Predictive value of preprocedural albuminuria for contrast-induced nephropathy non-recovery in patients undergoing percutaneous coronary intervention

**DOI:** 10.1007/s11255-021-02818-6

**Published:** 2021-03-06

**Authors:** Hanchuan Chen, Zhebin You, Sicheng Zhang, Chen He, Haoming He, Manjing Luo, Xueqin Lin, Liwei Zhang, Kaiyang Lin, Yansong Guo

**Affiliations:** 1 Department of Cardiology, Fujian Provincial Key Laboratory of Cardiovascular Disease, Fujian Provincial Hospital, Shengli Clinical Medical College of Fujian Medical University, Fuzhou, 350001 China; 2 Department of Geriatric Medicine, Fujian Key Laboratory of Geriatrics, Fujian Provincial Center for Geriatrics, Fujian Provincial Hospital, Fujian Medical University, Fuzhou, 350001 China

**Keywords:** Albuminuria, Contrast-induced nephropathy, Recovery, Percutaneous coronary intervention

## Abstract

**Objective:**

The present study investigated the predictive value of albuminuria for contrast-induced nephropathy (CIN) non-recovery in patients undergoing percutaneous coronary intervention (PCI).

**Methods:**

We retrospectively enrolled 550 consecutive patients inflicted with CIN after PCI and reassessing kidney function among 1 week–12 months between January 2012 and December 2018. Patients were stratified into three groups according to urine albumin: negative group (urine dipstick negative), trace group (urine dipstick trace) and positive group (urine dipstick ≥ 1 +). The primary outcomes were CIN non-recovery (a decrease of serum creatinine which remains ≥ 25% or 0.5 mg/dL over baseline at 1 week–12 months after PCI in patients inflicted with CIN). The odds ratio (OR) of CIN non-recovery was analyzed by logistic regression using the negative urine dipstick group as the reference group.

**Results:**

Overall, 88 (16.0%) patients had trace urinary albumin, 74 (13.5%) patients had positive urinary albumin and 40 (7.3%) patients developed CIN non-recovery. Patients with positive urinary albumin had significantly higher incidence of CIN non-recovery [negative (3.4%), trace (11.4%) and positive (23.0%), respectively; *P* < 0.0001]. Multivariate analysis showed that trace and positive urinary albumin were associated with an increased risk of CIN non-recovery (trace vs negative: OR 2.88, *P* = 0.022; positive vs negative: OR 2.99, *P* = 0.021). These associations were consistent in subgroups of patients stratified by CIN non-recovery risk predictors. And CIN non-recovery was associated with an increased risk of long-term mortality during a mean follow-up period of 703 days (*P* < 0.001).

**Conclusion:**

Preprocedural albuminuria was associated with CIN non-recovery in patients undergoing PCI.

## Introduction

Contrast-induced nephropathy (CIN), a common complication after coronary intervention procedure, has proved to be associated with poor short- and long-term outcomes [1]. However, the value of CIN is under heated debate [2, 3]. On one hand, most of it was temporary strike meaning limited effects on prognosis. On the other hand, it also confused the transient with persistent renal damage which makes the patients in mortal danger less distinguishable. Only when physicians identify the true high-risk patients who are susceptible to poor outcomes, they could take timely, economical and effective measures. The prognosis is not only determined on the severity of CIN but also the progression of renal recovery which was highlighted by several studies that indicated a very close association with the major adverse cardiovascular and nephropathic events. Therefore, we should remain vigilant at the renal function for a long postoperative period, identify the high-risk patients who are likely to develop CIN non-recovery and take necessary measures in time.

It is well established that albuminuria is useful in reflecting the development and mortality of the acute phase of kidney injury [4]. Moreover, it is also commonly accepted in the chronic phase of renal damage in the definition, staging, and prediction for outcome of chronic kidney disease (CKD) [5]. However, the association between albuminuria and CIN non-recovery remains unknown. Therefore, we sought to determine the predictive value of albuminuria for CIN non-recovery in patients undergoing percutaneous coronary intervention (PCI).

## Methods

### Study population

This is a retrospective observational study conducted at Fujian provincial hospital. Between January 2012 and December 2018, a total of 646 consecutive patients inflicted with CIN after PCI and reassessing kidney function among 1 week–12 months were enrolled. Exclusion criteria were as follows: (1) end-stage renal disease (eGFR < 15 mL/min/1.73m^2^) or long-term dialysis treatment (*n* = 10); (2) patients who died within 1 week after PCI (*n* = 25); (3) lack of data on SCr (*n* = 40); (4) cancer with expectation of life less than 1 year (*n* = 8); (5) use of contrast medium within the last 7 days (*n* = 7); (6) nonsteroidal anti-inflammatory drugs (NSAIDs) or other nephrotoxic drugs use within 48 h before the procedure (*n* = 6). (7) Patients who received IABP treatment (*n* = 0). Consequently, 550 patients were enrolled in this study.

### Definitions

CIN was defined as an absolute SCr increase 0.5 mg/dL or a relative increase in SCr ≥ 25% within 48 h after contrast medium exposure [6]. CIN non-recovery was defined as a relative decrease of serum creatinine which remains ≥ 25% or 0.5 mg/dL over baseline at 1 week–12 months after PCI in patients who developed CIN. Albuminuria was measured using a dipstick before procedure, we defined as follow: urine dipstick negative as “negative albuminuria”, urine dipstick trace as “trace albuminuria” and urine dipstick ≥ 1 + as “positive albuminuria” [7].

Hypotension was defined as systolic blood pressure (SBP) < 80 mmHg for at least 1 h requiring the support with medications or intra-aortic balloon pump (IABP) within 24 h peri-procedure [8].

The primary end point was the occurrence of CIN non-recovery. Additional end point was long-term mortality.

### Study protocol

SCr concentrations were measured on hospital admission before the procedure, every day for the following 2 days, at discharge, 1 week–12 months after discharge. Modified diet in renal disease (MDRD) formula was used to eGFR [9]. Routine urinalysis of fresh urine samples in morning which involved urinary albumin was measured before procedure and tested using an Urisys automatic analyzer. The 0.9% normal saline at a rate of 1 mL/kg/h was administered intravenously for approximately 12 h during the perioperative period (0.5 mL/kg/h if patients with heart failure). Relevant baseline, clinical data, and laboratory were recorded during the hospital. After discharge, all patients were measured routine urinalysis 1 week–12 months after discharge and subjected to follow-up for more than 1 year, which were monitored and recorded by nurses using clinical visits or telephone contact. This study was approved by the ethic committee of our institution and the subjects gave informed consent.

### Percutaneous coronary intervention

PCI was performed using standard techniques including standard guide catheters, guidewires, balloon catheters, and stents via the femoral or radial approach according to current guidelines [10]. The non-ionic, low-osmolar contrast media (either Iopamiron or Ultravist, both 370 mg I/mL) was used in all patients. The contrast dose, pharmacological therapies, IABP support were left to the discretion of cardiologists and patients’ condition.

### Statistical analysis

Statistical analysis was done with R version 4.0.2. Patients were stratified into three groups according to urinary albumin: negative group (urine dipstick negative), trace group (urine dipstick trace) and positive group (urine dipstick ≥ 1 +). Normally distributed continuous variables are reported as mean ± standard deviation (SD). Categorical data are expressed as absolute value and percentage. The Student’s *t* test, Wilcoxon rank sum test, or one-way analysis of variance was performed to determine the differences among groups. Categorical variables were compared by Chi-square test or Fisher exact test. Risk factors were identified on univariate logistic regression analysis included variables with *P* value < 0.05. Multivariable-logistic analysis was used to examine the association of positive group (urine dipstick ≥ 1 +) and trace group (urine dipstick trace) (vs negative group) with CIN non-recovery in models adjusted as follows: model 1 adjusted for age, eGFR < 90 mL/min/1.73m^2^, and model 2 adjusted for variables in model 1 plus diabetes mellitus, left ventricular ejection fraction (LVEF), anemia. Kaplan–Meier curve was used to compute the cumulative incidence of mortality stratified by urinary albumin levels and was compared using the log-rank test. A 2-sided *P* value < 0.05 was considered statistically significant.

## Results

### Baseline characteristics

A total of 550 consecutive patients were included. 161 (29.3%) were female, and the overall patient age was 65.3 ± 11.4 years. Baseline patient eGFR and SCr were 105 ± 31.2 mL/min/1.73m^2^ and 0.85 ± 0.67 mg/dL. Overall, 40 (7.3%) patients developed CIN non-recovery.

Table 1 shows the baseline characteristics of patients with and without CIN non-recovery. Patients with CIN non-recovery were older, more frequently to have anemia, diabetes, worse renal function, higher baseline of NT-proBNP, urine albumin levels, and lower LVEF.

**Table 1:** Baseline variables between CIN recovery group and non-recovery group

	CIN recovery (*n* = 510)	CIN non-recovery (*n* = 40)	*P* value
Demographics			
Age, years	64.9 ± 11.3	70.2 ± 11.3	0.006
Age > 75 years, *n* (%)	113 (22.2%)	14 (35.0%)	0.097
Sex, female, *n* (%)	146 (28.6%)	15 (37.5%)	0.314
Systolic blood pressure, mmHg	135.2 ± 22.6	130.4 ± 29.5	0.328
Diastolic blood pressure, mmHg	76.7 ± 14.0	74.9 ± 15.0	0.474
Medical history			
Hypertension, *n* (%)	357 (70.0)	33 (82.5)	0.135
Diabetes, *n* (%)	207 (40.6)	26 (65.0)	0.004
Atrial fibrillation, *n* (%)	50 (9.8)	7 (17.5)	0.170
Malignancy	6 (1.2)	2 (5.0)	0.109
Medical therapy during hospitalization			
Statin use, *n* (%)	509 (99.8)	40 (100)	1.000
CCB use, *n* (%)	160 (31.4)	13 (32.5)	1.000
Antiplatelet agents use, *n* (%)	505 (99.0)	38 (95.0)	0.086
β-blocker use, *n* (%)	412 (80.8)	35 (87.5)	0.402
Laboratory measurements			
WBC, 10^9^/L	8.7 ± 3.6	9.4 ± 3.0	0.193
Anemia, *n* (%)	124 (24.3%)	19 (47.5%)	0.002
PLT, 10^12^/L	222.8 ± 66.5	223.9 ± 66.5	0.926
Cholesterol, mmol/L	4.46 ± 1.24	4.53 ± 1.45	0.795
LDL-C, mmol/L	2.92 ± 1.10	2.91 ± 1.23	0.955
HDL-C, mmol/L	1.08 ± 0.29	1.11 ± 0.32	0.547
Urine albumin levels, *n* (%)			< 0.001
Negative	373 (73.5)	13 (32.5)	
Trace	78 (15.3)	10 (25.0)	
Positive	57 (11.2)	17 (42.5)	
Serum creatinine, mg/Dl	0.8 ± 0.5	1.5 ± 1.6	0.005
eGFR, mL/min/1.73 m^2^	107.6 ± 28.7	72.8 ± 42.2	< 0.001
eGFR < 90 mL/min/1.73 m^2^, *n* (%)	118 (23.1)	27 (67.5)	< 0.001
LVEF, %	56.2 ± 7.9	51.1 ± 8.7	0.002
Contrast volume, mL	191.8 ± 61.6	186.2 ± 61.9	0.587
Number of lesions, *n*	2.3 ± 0.8	2.6 ± 0.7	0.015
Number of stents, *n*	1.5 ± 0.8	1.7 ± 1.1	0.452

### CIN non-recovery incidences and risk factors

**Fig. 1:**
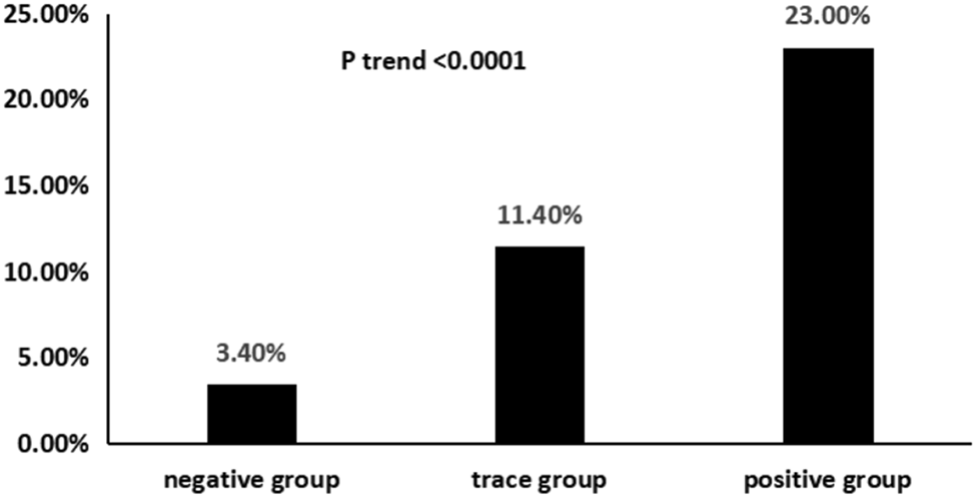
Incidence of CIN non-recovery

The incidences of CIN non-recovery were 3.40, 11.40, and 23.00% in patients in negative group, trace group and positive group (Fig. 1). Univariate logistic regression analysis indicated that age, eGFR < 90 mL/min/1.73m^2^, diabetes mellitus, LVEF and anemia were significantly associated with CIN non-recovery (all *P* < 0.05). In multivariable-adjusted logistic proportional hazard models, compared with negative group, trace group and positive group were both significantly associated with increased risk of CIN non-recovery, independent of demographics and clinical risk factors (Table 2). In model 1, after adjustment for age and eGFR < 90 mL/min/1.73m^2^, trace urinary albumin and positive urinary albumin were both significantly correlated with CIN non-recovery, and the odds ratios (OR) values were 3.44 (95% CI 1.38–8.33) and 5.66 (95% CI 2.49–13.06), respectively. In model 2, after adjusting for model 1 plus diabetes mellitus, LVEF, anemia, trace urinary albumin and positive urinary albumin remained associated with increased risk of CIN non-recovery, the OR values were 2.88 (95% CI 1.14–7.11, *P* = 0.022) and 2.99 (95% CI 1.17–7.56, *P* = 0.021), respectively.

**Table 2:** Associations between urinary albumin levels and CIN non-recovery

Urinary albumin	Participants, *n*	Events, *n*	Rate, %	Model 1^*^ OR (95% CI)	*P* value	Model 2^†^ OR (95% CI)	*P* value
Negative	388	13	3.40	1.00 (ref)		1.00 (ref)	
Trace	88	10	11.40	3.44 (1.38–8.33)	0.006	2.88 (1.14–7.11)	0.022
Positive	74	17	23.00	5.66 (2.49–13.06)	< 0.0001	2.99 (1.17–7.56)	0.021

*CI* confidence interval, *HR* hazard ratio

^*^Model 1 adjusted for age, eGFR < 90 mL/min/1.73m^2^; ^†^model 2 adjusted for variables in model 1 plus diabetes mellitus, LVEF, anemia

### Subgroup analysis based on CIN non-recovery risk predictors

**Fig. 2:**
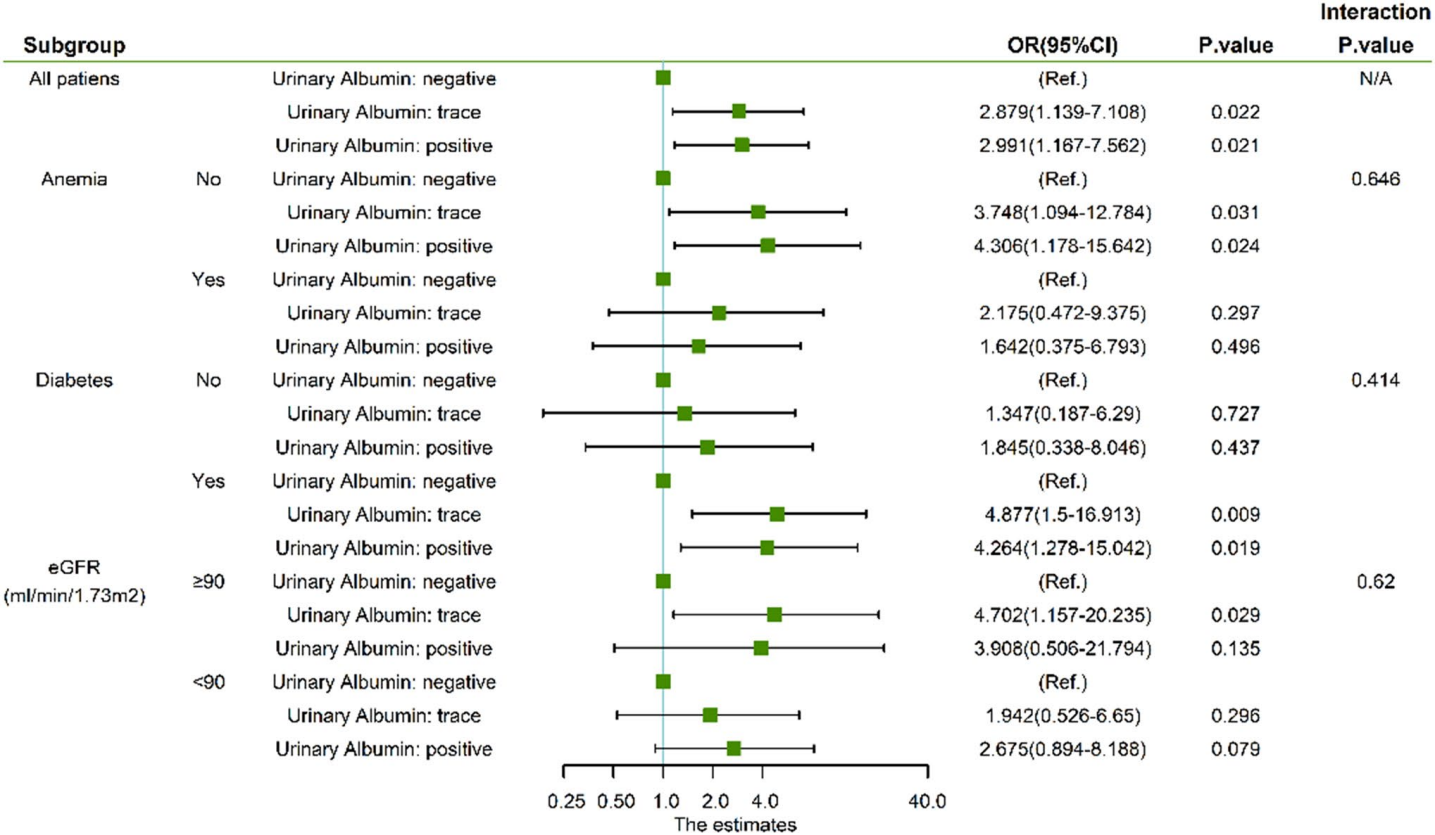
Forest and interaction

Figure 2 shows subgroup analysis stratified by CIN non-recovery risk factors, the association between urinary albumin levels and CIN non-recovery were consistent among these subgroups, there was no effect modification of anemia, diabetes and eGFR.

### Follow-up

**Fig. 3:**
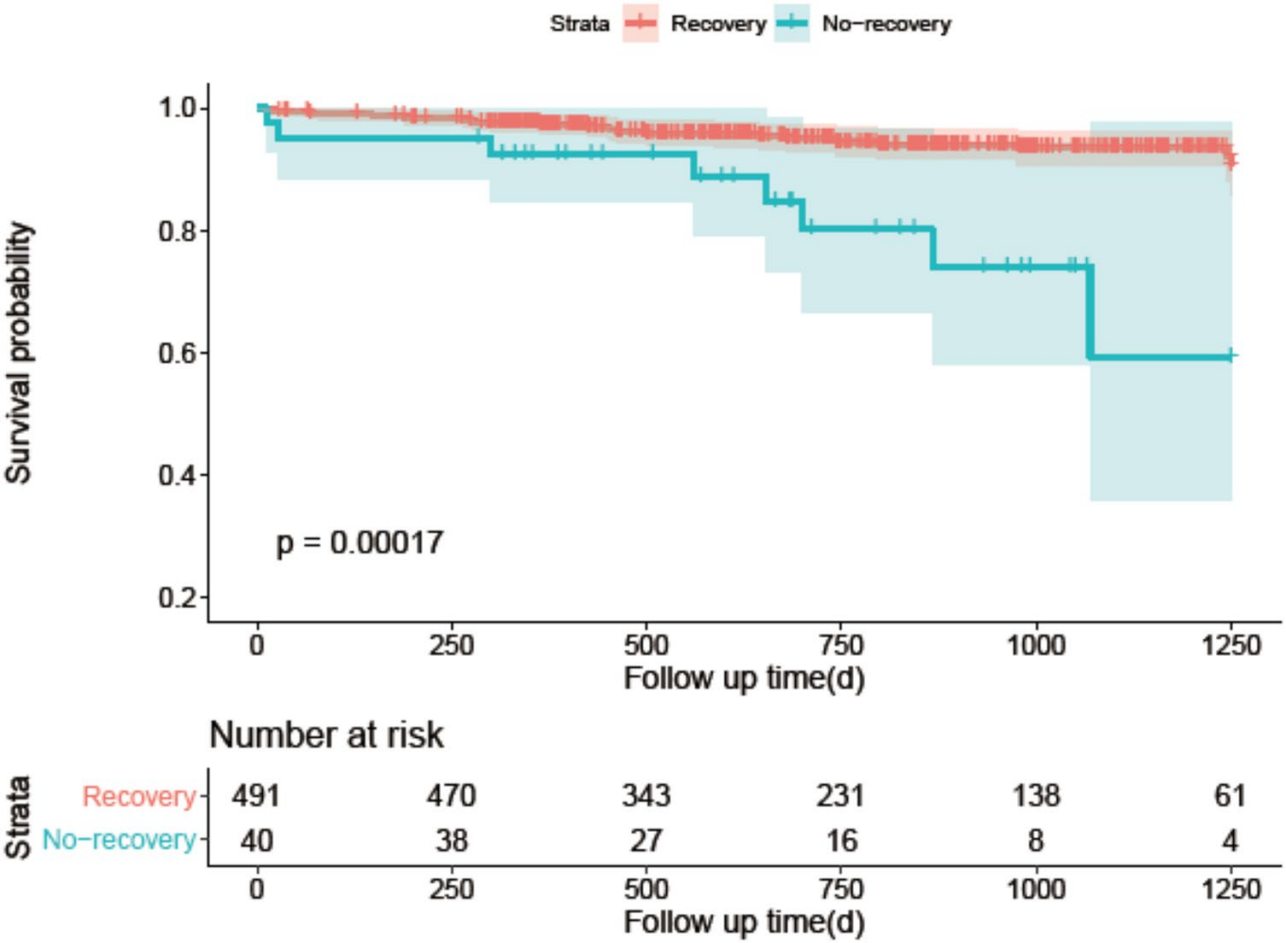
Mortality between CIN non-recovery

Median follow-up was 703 days (422–1060 days). Clinical outcomes were available for 531 patients (96.5%). 36 patients died, which represents 6.78% of all patients enrolled in our study. Kaplan–Meier curve demonstrated that patients suffering from CIN non-recovery presented high all-cause mortality (*P* < 0.001) (Fig. 3).

## Discussion

To our knowledge, this is the first study to demonstrate that preprocedural albuminuria was associated with CIN non-recovery in patients undergoing PCI. It showed that trace and positive urinary albumin were associated with an increased risk of CIN non-recovery (trace vs negative: OR 2.879, *P* = 0.022; positive vs negative: OR 2.991, *P* = 0.021) which was consistent among several subgroups of patients stratified by CIN non-recovery risk predictors. Furthermore, the occurrence of CIN non-recovery was associated with higher mortality (*P* < 0.001).

CIN, generally considered to rank the third among the causes of hospital-acquired kidney injury [11], could raise in-hospital mortality rate by about fivefolds and 1- and 5-year mortality rate about fourfolds [1]. It is contrary to the stereotype that CIN is always reversible and returns to baseline quickly, persistent renal damage occurred in 16.4–45.9% patients with CIN [12–14] and significantly increased the risk of adverse cardiovascular events and renal outcome compared with both patients with transient renal damage (*P* = 0.015) and without acute kidney injury (*P* = 0.0001) after coronary angiography [12]. Therefore, it is important to identify the high-risk patients with CIN developing into non-recovery and organize follow-ups so as to make more targeted preventive measures.

Multiple risk factors for CIN have been studied, from biomarker including NGAL, KIM-1, NAG to various clinical models such as Merhan risk score, Marenzi’s simple risk score, Mosquera’s 5 factor score etc. containing age, comorbidities, baseline renal function, hydration usage, contrast volume and so on [8, 15, 16]. The predictive value of CIN on long-term mortality is under heated debate. On the one hand, some studies doubted the role of CM in causing CIN, as they believed the latter may be overestimated for what is actually normal fluctuation of serum creatinine in hospital [17]. On the other hand, several researchers worried the confusion of transient kidney damage with long-term renal injury will mislead physicians in terms of real perniciousness of CIN who would underestimate the risk of CIN on prognosis. Unfortunately, only a few studies further drew a distinction between the two different patterns and evaluated classical injury markers for the purpose of predicting maladaptiveness or even the failure to recover.

Albuminuria is commonly accepted as an essential detection for the diagnostic, pathogenic, progression to ESRD and prognostic recommended by KDIGO guideline about CKD management [5]. Moreover, albuminuria was found to be an important risk factor for the development of AKI in patients after cardiac surgery. Molnar et al. demonstrated that individuals with the higher levels of perioperative dipstick albuminuria had the greatest risk for AKI (RR 2.46; 95% CI 1.16–4.97) and improved the clinical prediction of AKI above clinical models alone [18]. Recently, some researchers reported a close look at the predictive value of albuminuria for CIN after coronary angiography, the study of Meng et al. and Saito et al. suggested the preprocedural albuminuria was associated with greater risk for CIN regardless of the baseline renal function (eGFR < 30 mL/min/1.73m^2^: OR 17.4, *P* = 0.0001; eGFR 30–44 mL/min/1.73m^2^: OR 12.1; *P* = 0.0006; eGFR ≥ 60 mL/min/1.73m^2^: 12.1 vs 5.0%, *P* = 0.005) [19, 20]. For patients with T2DM undergoing elective cardiac catheterization, Yang et al. found preprocedural albuminuria was an independent risk factor of CIN (OR 3.8; 95% CI 1.5–9.2; *P* = 0.004) [7]. Their studies, however, did not attempt to assess the association of albuminuria and the long-term prognosis, let alone the repair process, also known as CIN non-recovery. To the best of our knowledge, there was not even a study focusing on the predictive value of albuminuria levels for CIN non-recovery. Our research filled the gap and provided that urine albumin level was a strong and independent predictor of CIN non-recovery after PCI even after adjusting for potential confounding factors. Meanwhile, the occurrence of CIN non-recovery was associated with higher mortality. In our study, the positive predictive value of albuminuria to CIN non-recovery was low (17%), but its negative predictive value was 96.6%, which also has important clinical significance. Use of tests with high negative predictive value (NPV) will reduce unnecessary interventions and hospitalization of patients. Furthermore, such a tests with high NPV provide necessary evidence to physicians, so that they can perform exams requiring contrast media more confidently.

The mechanisms underlying the association of albuminuria and CIN non-recovery remain uncertain. Potential pathophysiological assumptions as follows may help to understand the underlying relationship between urine albumin levels and maladaptive recovery after CIN. First, albuminuria, reflecting the overexcretion of albumin from the glomerulus or over-reabsorption of albumin in proximal tubular cells proved to be a marker of wounded kidney tissue including glomerular and tubular damage after chronic, sustained or acute injury [21, 22] resulting in the compromise of ability to tolerate hemodynamic changes and other nephrotoxic insults and was associated with the long-term adverse cardiovascular and renal outcomes [23]. Second, a high concentration of albumin in tubular itself may aggravate the damage after administration of CM leading to higher osmotic pressure, severer renal medullary hypoxia and renal fibrosis even worse which was common pathological manifestation of CKD [24]. Finally, in early non-recovery stage of CIN, or initial stage of CKD injury in other words, interstitial capillaries become increasingly permeable allowing that many plasma proteins that never reach the renal interstitium are able to do so and trigger an inflammatory response [25] via the upregulation of NF-κB or MCP-1 [26] and activate oxygen species (ROS) formation [27] leading to glomerulosclerosis.

## Limitations

Our study has several limitations. First, this study was observational from a single center with limited samples which may be affected by confounding and selection biases, calling for multiple centers and sufficient quantity of samples in the future. Second, the urine albumin levels were measured using semi-quantitative methods which were not accurate as quantitative methods. Third, considering the potential effects of two continuous stages of CIN recovery process may be a mixed factor, the definition of CIN non-recovery may not be accurate, but it also provides an essential detection in the follow-up period. Fourth, data of more detailed follow-up information about cardiovascular and renal outcomes were not recorded. Finally, we did not confirm our conclusion through biomarkers involved in fibrosis, inflammation, oxidative stress which will be left to the next stage of series of our researches.

## Conclusions

Preprocedural albuminuria was an independent risk factor for CIN non-recovery in patients undergoing PCI. As albuminuria is a convenient, rapid, cheap laboratory test that can be performed before PCI, it could help clinicians to identify the high-risk patients, take necessary measures and organize the follow-ups.

## Data Availability

The datasets used and/or analysed during the current study are available from the corresponding author on reasonable request.

## Abbreviations

CIN: Contrast-induced nephropathy
PCI: Percutaneous coronary intervention
SCr: Serum creatinine
BUN: Blood urea nitrogen
IABP: Intra-aortic balloon pump
LDL-C: Low-density lipoprotein cholesterol
LVEF: Left ventricular ejection fraction
eGFR: Estimated glomerular filtration rate
